# iLSGRN: inference of large-scale gene regulatory networks based on multi-model fusion

**DOI:** 10.1093/bioinformatics/btad619

**Published:** 2023-10-18

**Authors:** Yiming Wu, Bing Qian, Anqi Wang, Heng Dong, Enqiang Zhu, Baoshan Ma

**Affiliations:** School of Information Science and Technology, Dalian Maritime University, Dalian 116026, China; School of Information Science and Technology, Dalian Maritime University, Dalian 116026, China; Department of Statistics and Actuarial Science, The University of Hong Kong, Hong Kong 999077, China; School of Information Science and Technology, Dalian Maritime University, Dalian 116026, China; Institution of Computing Science and Technology, Guangzhou University, Guangzhou 510006, China; School of Information Science and Technology, Dalian Maritime University, Dalian 116026, China

## Abstract

**Motivation:**

Gene regulatory networks (GRNs) are a way of describing the interaction between genes, which contribute to revealing the different biological mechanisms in the cell. Reconstructing GRNs based on gene expression data has been a central computational problem in systems biology. However, due to the high dimensionality and non-linearity of large-scale GRNs, accurately and efficiently inferring GRNs is still a challenging task.

**Results:**

In this article, we propose a new approach, iLSGRN, to reconstruct large-scale GRNs from steady-state and time-series gene expression data based on non-linear ordinary differential equations. Firstly, the regulatory gene recognition algorithm calculates the Maximal Information Coefficient between genes and excludes redundant regulatory relationships to achieve dimensionality reduction. Then, the feature fusion algorithm constructs a model leveraging the feature importance derived from XGBoost (eXtreme Gradient Boosting) and RF (Random Forest) models, which can effectively train the non-linear ordinary differential equations model of GRNs and improve the accuracy and stability of the inference algorithm. The extensive experiments on different scale datasets show that our method makes sensible improvement compared with the state-of-the-art methods. Furthermore, we perform cross-validation experiments on the real gene datasets to validate the robustness and effectiveness of the proposed method.

**Availability and implementation:**

The proposed method is written in the Python language, and is available at: https://github.com/lab319/iLSGRN.

## 1 Introduction

The interaction between genes forms the dynamic biochemical network known as the gene regulatory networks (GRNs). Clarifying the biological mechanism of the cell cycle, damage repair, and apoptosis depends on understanding the information transmission in the biological GRN from a systematic perspective (Pataskar and Tiwari 2016, Jackson *et al.* 2020, Razaghi-Moghadam and Nikoloski 2020). In addition, GRNs can obtain the key regulatory genes related to cell diseases, which is conducive to the diagnosis and treatment of cancer and other complex diseases as well as the research and development of targeted drugs (Emmert-Streib *et al.* 2014, Mi *et al.* 2019, Oulas *et al.* 2019). The investigation on GRNs has great significance to the realization of precision medicine (Madhamshettiwar *et al.* 2012, Yan *et al.* 2016, Delgado-Chaves *et al.* 2020). With the advance of biological sequencing technology (Marbach *et al.* 2012, Barrett *et al.* 2013, Buermans and den Dunnen 2014), professionals have developed various feasible methods to reconstruct GRNs from gene expression data (Schaffter *et al.* 2011, Gama-Castro *et al.* 2016, Mercatelli *et al.* 2020). Some methods also incorporated different types of data (steady-state data, time-series data, etc.) in gene network inference (Bonneau *et al.* 2006, Werhli and Husmeier 2007, Greenfield *et al.* 2013, Petralia *et al.* 2015, Castro *et al.* 2019).

The methods based on information theory are widely used to infer GRNs (Margolin *et al.* 2006, Faith *et al.* 2007, Zhang *et al.* 2013). These methods including Pearson correlation coefficient, mutual information (MI), conditional mutual information, and Maximal Information Coefficient (MIC), apply a threshold to determine whether MI between the expression responses of two genes is sufficient to infer a connecting regulatory link (Stuart *et al.* 2003). The prominent advantage of MI is that it can identify the non-functional regulatory relationship between genes. Yang *et al.* proposed a method MICRAT (Maximal Information coefficient with Conditional Relative Average entropy and Time-series mutual information) based on MIC to infer GRN (Yang *et al.* 2018). The MIC provides a more general framework for measuring the dependence between two genes.

Model-based methods usually establish mathematical models to infer the regulatory relationship between genes, including Boolean network model, Bayesian network model, Neural network model, and Differential equation model. The Boolean network method adopts 0/1 value to represent gene expression level, and logic function to represent the relationship between genes (Saadatpour and Albert 2013). But the unreasonable threshold selection in the Boolean model will increase the noise, making the GRNs inaccurate (Chai *et al.* 2014). The Dynamic Bayesian network takes time-series data as input, which enables the Bayesian model to effectively utilize the time information in gene expression data (Kim *et al.* 2003, Li *et al.* 2014, Sanchez-Castillo *et al.* 2018). The Bayesian model tends to suffer from a long-running time for analyzing large-scale genomic data, which could be resolved by the Local Bayesian network model (Liu *et al.* 2016). A highly flexible and scalable method, the neural network model, which simulates different functional relationships can represent the dynamic relationship of genes (Vohradsky 2001, Yuan and Bar-Joseph 2019). The different neural network models could be used to process different types of gene data, such as convolutional neural networks for gene expression images (Yang *et al.* 2019) and recurrent neural networks for gene time-series data (Ressom *et al.* 2006). The differential equation model employs a differential equation to construct a model to describe the relationship between genes, which is mainly divided into the linear ordinary differential equations (ODEs) method (Matsumoto *et al.* 2017, Fan *et al.* 2019) and the non-linear ODEs method (Ma *et al.* 2020, Tsai *et al.* 2020). Ma *et al.* proposed a Non-linear ODEs inference algorithm to establish a dynamic gene regulatory model and utilized importance scores to effectively infer all regulatory relationships among genes (Ma *et al.* 2020). Henderson *et al.* developed a non-parametric additive differential equation model to process time-series data (Henderson and Michailidis 2014). The advantage of differential equation is that it can be exploited to analyze both steady-state and time-series data (Delgado and Gómez-Vela 2019).

Machine-learning-based methods mainly apply machine-learning models and data structures to fit gene expression data. The GENIE3 method is model-free for inferring GRNs from steady-state series gene expression data (Huynh-Thu *et al.* 2010). However, it cannot be exploited for time-series data. The dynGENIE3 adapts the GENIE3 method to process time-series and steady-state series gene expression data (Huynh-Thu and Geurts 2018). Zheng *et al.* proposed the BiXGBoost method to solve the over-fitting problem by integrating randomization and regularization techniques (Zheng *et al.* 2019). The MMFGRN combines three models: a single time-series data model, a single steady-data model, and a single time-series and steady-state series data joint model (He *et al.* 2021). The results of MMFGRN imply that the model fusion strategy is feasible for GRN prediction. These methods are highly explanatory and can identify the regulatory direction, so the GRN obtained is often a directed network.

Currently, different inference algorithms have made great achievements, especially for small-scale GRNs. However, for large-scale networks, the high dimensionality, sparsity, and non-linearity of the network result in a significant decline in the efficiency and accuracy of inference algorithms (Fan *et al.* 2019, Ma *et al.* 2020). Here, we initially employ MIC to shrink the regulatory gene’s dimension for the given gene expression data. The next step is to infer GRNs using XGBoost and RF models. The machine-learning algorithms can efficiently capture gene regulatory relationships from both time-series data and steady-state series data. The results using the DREAM4 and *Escherichia coli* datasets demonstrate that our method performs better compared with state-of-the-art methods when inferring large-scale GRNs.

## 2 Datasets

In this article, we take the DREAM4 *in silico* size100 simulated data and *E.coli* real gene expression data as our experimental datasets. Table 1 shows the details of the datasets we used.

**Table 1.** Datasets.^a^

Network	DREAM4 *in silico* size100		*Escherichia coli*			
	Time-series data	Steady-state series	Cold stress	Heat stress	Oxidative stress	Lactose
Genes	100	100	3959	3959	3959	3959
Samples	10	200	3	3	3	3
Time points	21		8	8	11	5
TFs	100	100	114	114	114	114

a This table contains two datasets, DREAM4 and *Escherichia coli*, and introduces the number of genes, samples, time points, and regulatory factors in the dataset. DREAM4 contains five subnets, and the *Escherichia coli* dataset contains gene expression data under four different environments.

### 2.1 DREAM4 *in silico* size100 dataset

The DREAM4 *in silico* size100 dataset is a simulated gene expression data provided by Gene Net Weaver (GNW). The GNW is widely used for the inference of GRNs, including three public inferential GRN competitions, DREAM5, DREAM4, and DREAM3 (Schaffter *et al.* 2011). The *in silico* size100 incorporates both time-series data and steady-state series data. The time-series data contains 10 samples of 100 genes, and each sample has 21 time points. Steady-state series data includes gene expression levels under different perturbations.

### 2.2 *Escherichia coli* dataset

Real gene expression data of the *E.coli* dataset is collected from the GEO database (GSE20305), which records the expression levels of about 4400 genes under five different environmental perturbations (cold, heat, oxidative stress, lactose diauxie, and stationary phase) (Jozefczuk *et al.* 2010). The gold standard network in the RegulonDB provides experimentally validated regulatory relationships among genes (Gama-Castro *et al.* 2016). After matching the *E.coli* dataset with the gold standard network and retaining the common genes, the dataset contains 3959 genes and 114 TFs (Ma *et al.* 2020).

## 3 Materials and methods

**Figure 1.**
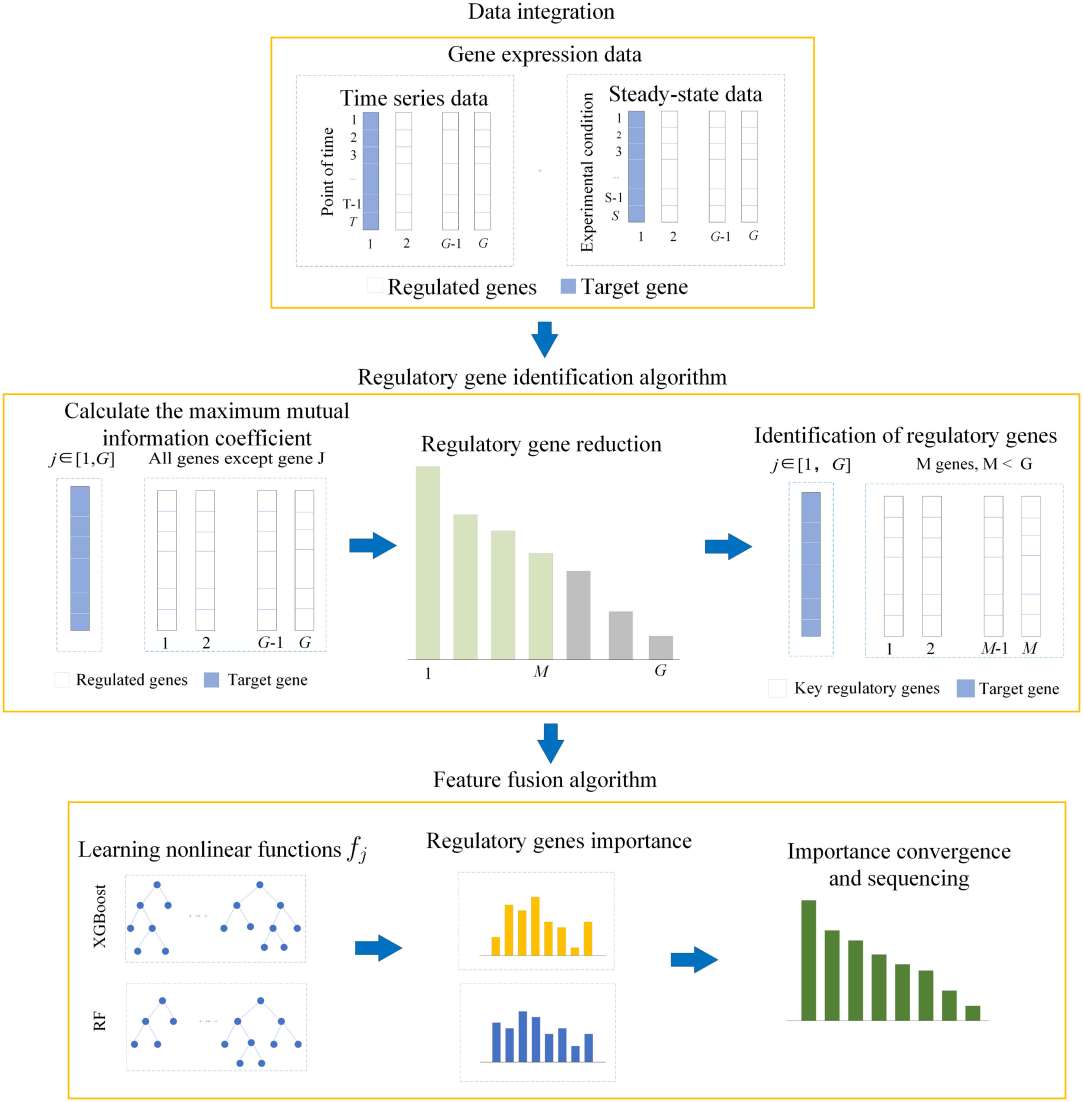
Overall flow chart of the iLSGRN. The overall process mainly consists of three parts: data integration, regulatory gene identification algorithm, and feature fusion algorithm

The overall flow of our method is shown in Fig. 1. Assuming that there are *G* genes in total, we first combined time-series data with steady-state series data. Then, we removed the redundant gene regulatory links using the regulatory gene identification algorithm to reduce the dimension of large-scale gene data. The MIC has a good ability to identify regulatory relationships between genes, so we applied MIC to construct the regulatory gene identification algorithm (Yang *et al.* 2018). After shrinking the candidate regulatory genes of target gene *j* (*j *=* *1,2,…,*G*), we obtained *M* regulatory genes (*M≪G*). In the feature fusion algorithm, we applied a non-linear ODEs model to describe gene regulatory relationship behaviors. The non-linear ODEs are more suitable for simulating the dynamic characteristics of genes and processing the time-series data and steady-state series data. Then, the XGBoost and RF are used to train the non-linear ODEs model, respectively, and the importance scores derived from the above two models are fused to obtain the final gene regulatory relationship.

### 3.1 Non-linear ODE model

We employ a non-linear ODEs to construct gene networks. We define the non-linear ODEs model for time-series data as follows:


(1) $$\frac{dx_{t}^{j}}{dt}+c_{j}x_{t}^{j}=f_{j}\left(x_{t}^{i}\right),i=\left\{1,2,\ldots ,M\right\},$$

where $x_{t}^{j}$ is the expression value of target gene *j* at time *t*, $c_{j}$ is decay rate, $x_{t}^{i}$ is a vector containing all regulatory gene expression levels at time *t*, and $f_{j}$ represents the non-linear function of *M* regulatory genes with respect to target gene *j*. Furthermore, for discrete time-series data, we utilize the difference equation to approximate the differential equation and simplify Formula 1 to:


(2) $$\frac{x_{t_{k+b}}^{j}-x_{t_{k}}^{j}}{t_{k+b}-t_{k}}+c_{j}x_{t_{k}}^{j}=f_{j}\left(x_{t_{k}}^{i}\right),k=1,2,\ldots ,T,$$

where *b* is the time step, the default value of *b* is 1. For steady-state series data, we define $\frac{{dx}_{t}^{j}}{dt}=0$. Then, the non-linear ODEs model for steady-state series data is simplified to Equation (3). Where *e* denotes different experimental conditions.


(3) $$c_{j}x_{e}^{j}=f_{j}\left(x_{e}^{i}\right),e=1,2,\ldots ,S.$$

### 3.2 Regulatory gene identification algorithm

According to the sparsity of large-scale GRNs, we proposed a regulatory gene identification algorithm. The approach calculates the correlation between the target gene and all candidate regulatory genes using MIC. The prediction accuracy of the proposed approach can be improved by identifying the most important regulatory genes. Moreover, our algorithm can effectively shrink potential candidate genes and avoid massive computational costs in the following training step of the machine-learning model.

We define the MI between gene *x* and gene *y* as:


(4) $$I\left(X;Y\right)=\sum_{y\in Y}\sum_{x\in X}p\left(x,y\right)\log_{2}\left(\frac{p\left(x,y\right)}{p\left(x\right)p\left(y\right)}\right),$$

(5) $$\mathrm{MIC}\left(X;Y\right)=\max\{\frac{I\left(X;Y\right)}{\log_{2}\;\min\left(m,n\right)}\},\;$$

where *X* and *Y* represent the expression sequences for gene *x* and *y*, respectively. $p\left(x,y\right)$ is the joint probability distribution function of *X* and *Y*, and $p\left(x\right)$ and $p\left(y\right)$ denote the marginal probability distribution functions of *X* and *Y*. Generally, the plane including *x* and *y* is divided into grids to calculate MIC, m,n are the numbers of horizontal and vertical grids, respectively, $m\times n<\mathrm{data}\_\mathrm{siz}e^{0.6}$, and $\log_{2}\;\min\left(m,n\right)$ is used to normalize the MIC (Yang *et al.* 2018).

For *G* genes in the whole gene expression dataset, gene *j* is selected as the target gene, and the remaining genes are chosen as candidate regulatory genes. We calculate the MIC between a given target gene *j* and a candidate regulatory gene, exclude redundant regulatory genes according to the threshold of MIC, and attain the regulatory gene sets $R_{j}$ for the target gene *j*. After repeating the above steps to obtain the regulatory gene set $R=\left\{R_{1},\ldots ,R_{j}\ldots ,R_{G}\right\}$ for all target genes, our algorithm achieves the goal of regulatory gene identification.

### 3.3 Feature fusion algorithm

The feature fusion method employs XGBoost and RF models to learn the function $f_{j}$. At present, these two advanced machine-learning algorithms support parallel computing and can substantially enhance the efficiency of the fusion algorithm. After obtaining the regulatory gene $R_{j}$ for target gene *j*, we adopt machine-learning algorithms to learn the non-linear function $f_{j},\;$respectively, and calculate the importance scores between regulatory genes and target gene *j*. The regulatory gene importance list for target gene *j* is calculated based on geometric mean method as follows:


(6) $$\mathrm{Scor}e_{j}=\mathrm{ScoreXGB}\cdot \mathrm{ScoreRF},$$

where ScoreXGB is the regulatory gene importance list of target gene *j* derived by the XGBoost model. ScoreRF is the regulatory gene importance list of target gene *j* derived by the RF model. We multiply these two vectors to generate the final regulatory gene importance list for target gene *j*. After repeating the above steps to obtain the importance lists for all target genes, we finally integrate the importance lists into the importance matrix, which shows the regulatory relationship between any two genes.

### 3.4 Evaluation method

Usually, a confusion matrix is applied to evaluate the accuracy of the inference algorithm, including the number of true positive (TP) samples, false negative (FN) samples, false positive (FP) samples, and true negative (TN) samples. Based on the confusion matrix, four evaluation indicators including recall, precision, true positive rate (TPR), and false positive rate (FPR), could be further calculated.


(7) $$\mathrm{Recall}=\frac{\mathrm{TP}}{\mathrm{TP}+\mathrm{FN}},$$

(8) $$\mathrm{Precision}=\frac{\mathrm{TP}}{\mathrm{TP}+\mathrm{FP}},$$

(9) $$\mathrm{TPR}=\frac{\mathrm{TP}}{\mathrm{TP}+\mathrm{FN}},$$

(10) $$\mathrm{FPR}=\frac{\mathrm{FP}}{\mathrm{FP}+\mathrm{TN}}.$$

This study applies two evaluation metrics, area under precision–recall curves (AUPR) and area under receiver operating characteristic (AUROC), to assess the performance of the inference methods for GRNs. The AUROC and AUPR scores can better reflect the comprehensive performance of the classification algorithms. And we also define an overall score to evaluate the performance of the different methods.


(11) $$\text{Overall Score}=\;\frac{\mathrm{AUROC}+\;\mathrm{AUPR}}{2}.$$

We also consider the early precision ratio (EPR) to evaluate the accuracy of the top *k* edges of the inferred network. And *k* denotes the number of edges with the label “1” in the gold standard network. Early precision (EP) refers to the fraction of TPs in the top *k* edges, and the EPR represents the ratio of the EP of the proposed model to a random predictor, where a random predictor’s EP is the edge density. The edge density is defined as the ratio of edges with the label “1” to all potential edges in the gold standard network, and the network of *n* genes has $n\times \left(n-1\right)$ potential edges (Pratapa *et al.* 2020).


(12) $$\mathrm{EP}=\frac{\left(\mathrm{top}\;k\right)\;\mathrm{TP}}{k},$$

(13) $$\mathrm{EPR}=\frac{\mathrm{EP}}{k/n\times (n-1)}.$$

### 3.5 Pseudocode flow chart

Algorithm 1 is the pseudo code flow chart of our method. Our algorithm takes the gene expression time-series data and steady-state series data as input, and the regulatory importance list for all genes as output.

Algorithm 1: The proposed algorithm for solving GRNs inference

**Input:** time-series data $X_{\mathrm{TS}}\left(k=1:T\right)$, including *G* genes and *T* time sampling points, steady sequence data $X_{s}\left(e=1:S\right)$, including *G* genes and *S* experimental conditions, threshold $T_{\mathrm{MIC}}$.

**Output:** gene regulatory network $w\in R^{G\;\times \;\left(G-1\right)}$.

1 Initialize w=0.

2 Calculate MIC coefficient.

3 for *j = 1*; *j ≤ G*; *j + +* do

4    for *i ≠ j*; *i ≤ G*; *i++* do

5            Combining time-series samples with steady-state series samples, the input and output pairs of training samples are obtained.

${\;\;\;\;\;\;\;\;\;X}^{j}=X_{TS}^{j}\cup X_{s}^{j}$.

${\;\;\;\;\;\;\;\;\;X}^{i}=X_{TS}^{i}\cup X_{s}^{i}$.

6    Dimensionality reduction based on MIC

         if $T_{\mathrm{MIC}}$ < MIC ($X^{j}$, $X^{i}$)

          Retain gene *i* to list $M_{j}$.

         else

          skip.

    Obtain all regulatory genes $R_{j}$ of target gene *j* from list $M_{j}$.

7    Build a non-linear ODEs model $f_{j}$. Using XGBoost and RF machine-learning model to calculate importance score ScoreXGB and ScoreRF.

8    Model importance score fusion.

 $\;\;\;\;\mathrm{Scor}e_{j}=\mathrm{ScoreXGB}\cdot \mathrm{ScoreRF}$.

9 All importance scores are obtained after ranking all importance.

10 Return $w_{i,j}\left(i\neq j\right),i,j=1,\ldots ,G$.

### 3.6 Related methods for inference of GRN

In this article, we make a comparison experiment of iLSGRN with other mainstream methods, including GENIE3, dynGENIE3, BiXGBoost, Non-linear ODEs, and MMFGRN. GENIE3 and dynGENIE3 utilize importance scores from RF to identify regulators for each target gene, and dynGENIE3 is an adapted version of the GENIE3 to deal with time-series and steady-state series data. BiXGBoost is based on the bidirectional model, and considers both candidate regulatory genes and target genes for a specific gene and uses XGBoost to calculate feature importance as regulatory gene relationships. Non-linear ODEs method combines non-linear ODE models and XGBoost to infer GRNs. MMFGRN fuses three models to infer GRNs through a weighted fusion strategy, including a time-series data model based on LightGBM and a steady-state data model based on LightGBM, as well as a time-series and steady-state data joint model based on XGBoost.

### 3.7 Parameter setting and cross-validation experiment

When setting the MIC thresholds in our method, we referred to the results in a previous study, which also used MIC to infer GRNs. In the study (Yang *et al.* 2018), a suitable MIC threshold (0.15) on the DREAM4 *in silico* size100 dataset was recommended. We set several values within a small neighboring range (0.1–0.2) of this threshold to infer the GRNs and chose the one that corresponded to the optimal result. Supplementary Figs S1 and S4 show that the MIC distributions on the *E.coli* dataset are significantly wider than those on the DREAM4 dataset, and the previous study has reported that the GRN of *E.coli* is basically very sparse (Fan *et al.* 2019), thus, we searched for the optimal threshold in a relatively large range (0.2–0.7) to exclude more redundant regulatory relationships on the *E.coli* dataset. Since this parameter setting method may cause overfitting, we further conducted cross-validation experiments to validate the robustness of the proposed method.

In the cross-validation experiment for the iLSGRN, we divided the genes into 2-folds, the training set and the test set. Here, we mainly discuss the effect of threshold and learning rate in XGBoost, and use the grid search method to adjust the parameters. Specifically, we set 10 different learning rates of XGBoost and 30 different thresholds of MIC on one training set, conducted a total of 300 experiments and recorded the overall score for each experiment. Next, the proposed model with the optimal parameters was applied on the test set. Finally, we switched the training set and test set and repeated the above-mentioned steps. The performance of the prediction algorithm is estimated by averaging the accuracy results of the two test sets.

In addition, we used the grid search method to search the optimal parameters for other inference methods in comparison experiments. For GENIE3 and dynGENIE3, the parameters “max_depth” and “n_estimators” were selected to optimize. For Non-linear ODEs and BiXGBoost, the optimized parameters include: “learning_rate, max_depth, n_estimators.” In MMFGRN, we optimized the parameters “learning_rate” in XGBoost and “learning_rate” in LigthGBM.

## 4 Results

In this section, we extensively compared our method with five state-of-the-art methods: Non-linear ODEs (Ma *et al.* 2020), GENIE3 (Huynh-Thu *et al.* 2010), dynGENIE3 (Huynh-Thu and Geurts 2018), BiXGBoost (Zheng *et al.* 2019), and MMFGRN (He *et al.* 2021). A comparative experiment was first conducted on the DREAM4 *in silico* size100 and the *E.coli* gene expression dataset. Then, we performed cross-validation and ablation experiments to verify the robustness and stability of the method. There is no denying that the decay rate is still an important factor. However, it is difficult to set an exact decay rate value for all genes, and we consider the decay rate as a constant.

### 4.1 Performance evaluation on DREAM4 *in silico* size100 dataset

In this part, we performed experiments of the above-mentioned approaches on the five sub-datasets of the DREAM4 dataset containing time-series data and steady-state series data. The main parameters of the iLSGRN on DREAM4 *in silico* size100 dataset are shown in Supplementary Table S1. Table 2 summarizes the results of these GRN inference methods on the DREAM4 *in silico* size100 dataset. The distribution of the MIC is provided by Supplementary Fig. S1. The AUROC curves of iLSGRN are provided by Supplementary Fig. S2. The iLSGRN achieves the highest AUROC in Networks 1 and 5 and the highest AUPR in all networks. Although the AUROC scores of the iLSGRN do not reach the best in Networks 2–4, our method is still more competitive than other inference methods in most subnets. The Cluster Bar Chart of the results for various methods on each sub-dataset of the DREAM4 *in silico* size100 is shown in Supplementary Fig. S3. The iLSGRN achieves the best overall score on all sub-datasets in Supplementary Fig. S3. The EPR results on DREAM4 dataset for each method are shown in Supplementary Table S2. The EPR values of iLSGRN on the five sub-networks are 29.08, 13.57, 20.83, 18.01, and 19.40, respectively, which are higher than other inference methods.

**Table 2.** The results of various methods on DREAM4 *in silico* size 100 dataset.

Methods		Net1	Net2	Net3	Net4	Net5
GENIE3	AUROC	0.825	**0.783**	**0.813**	**0.826**	0.791
	AUPR	0.129	0.119	0.147	0.150	0.113
dynGENIE3	AUROC	0.843	0.746	0.803	0.793	0.824
	AUPR	0.236	0.144	0.227	0.221	0.158
BiXGBoost	AUROC	0.806	0.730	0.765	0.735	0.769
	AUPR	0.235	0.152	0.261	0.204	0.214
Non-linear ODEs	AUROC	0.857	0.758	0.795	0.787	0.798
	AUPR	0.370	0.213	0.322	0.318	0.294
MMFGRN	AUROC	0.854	0.772	0.792	0.701	0.747
	AUPR	0.371	0.215	0.328	0.319	0.337
iLSGRN	AUROC	**0.859**	0.771	0.799	0.800	**0.839**
	AUPR	**0.462**	**0.262**	**0.389**	**0.357**	**0.342**

The best results for AUROC and AUPR scores are in bold.

### 4.2 Performance evaluation on *E.coli* dataset

Here, we apply the iLSGRN and other inference methods on the *E.coli* dataset. The main parameters of the iLSGRN on *E.coli* dataset are shown in Supplementary Table S3. Table 3 summarizes the performance of these methods on the *E.coli* dataset. As seen in Table 3, the iLSGRN obtains the best AUROC scores on Cold Stress, Heat Stress, and Oxidative stress sub-datasets, and the best AUPR scores on Cold Stress, Oxidative stress, and Lactose sub-datasets. MMFGRN achieves the best AUPR score on the Heat Stress subset and the best AUROC score on the Lactose subset. Overall, our method still maintains a high prediction accuracy and is competitive for reconstructing large-scale GRNs. Supplementary Fig. S4 shows the distribution of the MIC on the *E.coli* dataset and Supplementary Fig. S5 shows the AUROC curves of iLSGRN for each subset of *E.coli* data. Supplementary Fig. S6 is the cluster bar chart of the results for each method in comparison experiment. The EPR results on *E.coli* dataset for each method are shown in Supplementary Table S4. On the *E.coli* dataset, the iLSGRN obtains the highest EPR values on Cold stress, Oxidative stress, and the same EPR value as MMFGRN on Lactose.

**Table 3.** The results of various methods on *E.coli*.

Methods	Cold stress		Heat stress		Oxidative stress		Lactose	
	AUROC	AUPR	AUROC	AUPR	AUROC	AUPR	AUROC	AUPR
GENIE3	0.522	0.016	0.547	0.020	0.579	0.035	0.528	0.019
BiXGBoost	0.470	0.013	0.523	0.015	0.503	0.012	0.505	0.011
Non-linear ODEs	0.552	0.019	0.558	0.017	0.605	0.027	0.583	0.018
MMFGRN	0.689	0.023	0.812	**0.044**	0.762	0.038	**0.833**	0.035
iLSGRN	**0.784**	**0.026**	**0.878**	0.032	**0.803**	**0.059**	0.753	**0.036**

The best results for AUROC and AUPR scores are in bold.

### 4.3 Performance of the cross-validation experiments on *E.coli* dataset

To evaluate the robustness of the proposed method, we conducted 2-fold cross-validation experiments on the *E.coli* dataset. The main parameters of iLSGRN in the cross-validation experiment are shown in Supplementary Table S5. The cross-validation experimental results are shown in Table 4 and Supplementary Figs S7 and S8. In Table 4, iLSGRN has the highest scores except for the AUROC on the Cold Stress sub-dataset. Supplementary Fig. S7 shows the boxplot of overall score and threshold for *E.coli* dataset. Supplementary Fig. S8 is the cluster bar chart of the cross-validation results on the *E.coli* dataset. Although our method does not achieve the best scores in certain sub-datasets, our method still outperforms other methods, indicating the great potential of iLSGRN for inferring large-scale GRNs.

**Table 4.** The results of the 2-fold cross-validation experiments on *E.coli*.

Method	Cold stress		Heat stress		Oxidative stress		Lactose	
	AUROC	AUPR	AUROC	AUPR	AUROC	AUPR	AUROC	AUPR
GENIE3	0.441	0.020	0.531	0.015	0.409	0.028	0.515	0.017
BiXGBoost	0.442	0.011	0.510	0.013	0.517	0.005	0.467	0.012
Non-linear ODEs	0.491	0.016	0.512	0.017	0.557	0.021	0.569	0.014
MMFGRN	**0.572**	0.018	0.425	0.020	0.541	0.033	0.463	0.015
iLSGRN	0.571	**0.023**	**0.635**	**0.065**	**0.694**	**0.082**	**0.563**	**0.019**

The best results for AUROC and AUPR scores are in bold.

### 4.4 Results of the ablation experiment

**Figure 2.**
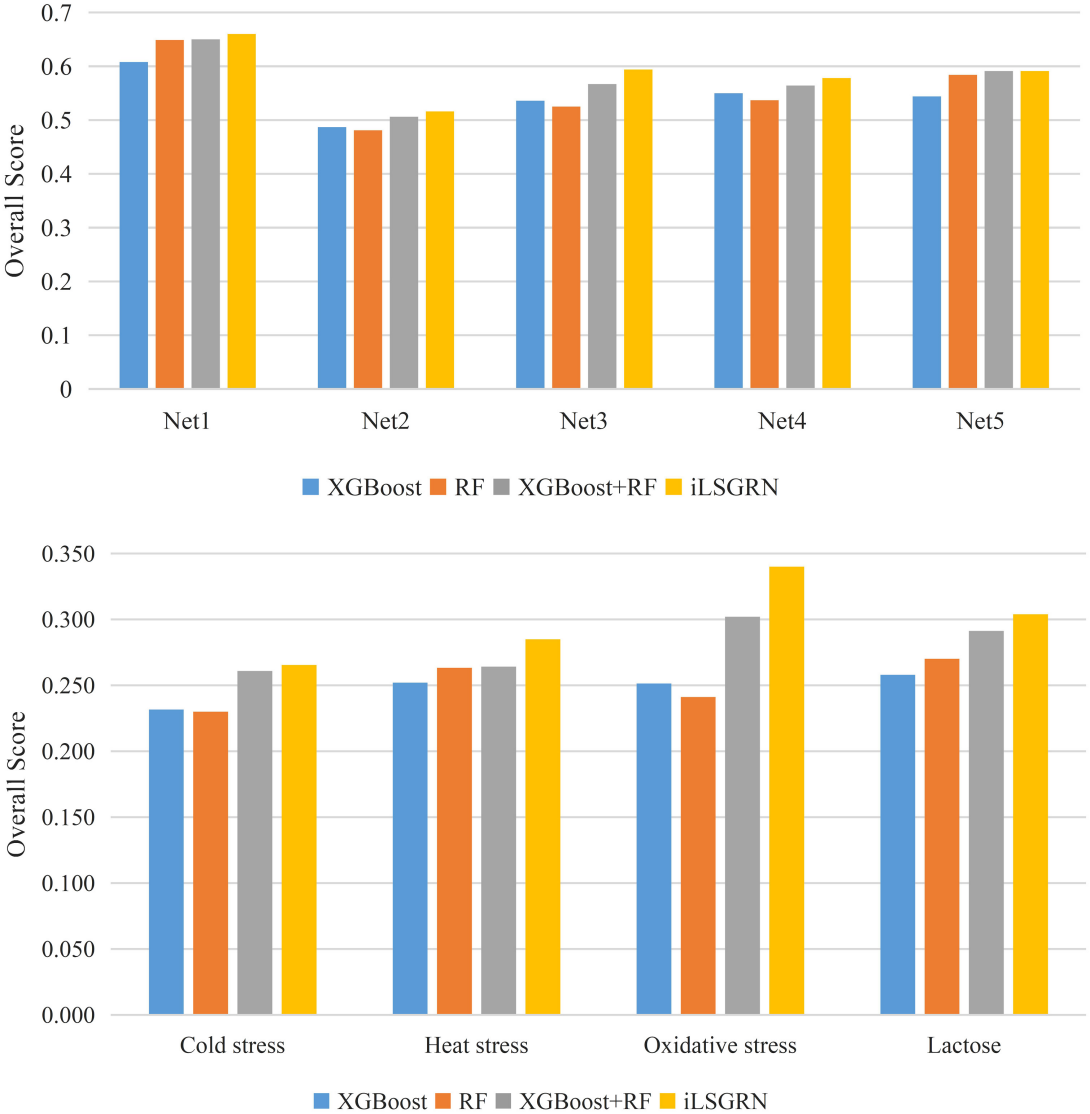
Cluster Bar Chart of the ablation experiment’s overall score on the DREAM4 *in silico* size100 and *E.coli* datasets

We performed ablation experiments on the DREAM4 *in silico* size100 and *E.coli* datasets to validate the effectiveness of each module in the proposed method. In the ablation experiments, the effects of our regulatory gene recognition algorithm and feature fusion algorithm can be evaluated systematically, as shown in Fig. 2. The “RF” represents only applying the RF algorithm to calculate the importance score. The “XGBoost” indicates only applying the XGBoost algorithm to calculate the importance score. The “XGBoost+RF” means combining the XGBoost with RF without the regulatory gene recognition algorithm.

The iLSGRN achieves the highest overall scores on all sub-datasets compared to the single-model approaches. From Fig. 2, we observed that the overall score gradually increases with the integration of the three single models. The results of the ablation experiments show that the regulatory gene recognition algorithm and the feature fusion algorithm have great advantages in gene network prediction.

### 4.5 Computational complexity

The iLSGRN algorithm is implemented based on XGBoost and RF. The computational complexity of XGBoost is on the order of O(KPdnlog⁡ n), where K is the number of trees, P indicates the number of genes, d represents the maximum depth of trees, and n means the total number of samples. And the computational complexity of RF is on the order of O(c×k), where c is the maximum depth of trees and k denotes the number of trees. We further compared the computational complexity of iLSGRN with GENIE3, BiXGBoost, Non-linear ODEs, and MMFGRN. Among these methods, BiXGBoost utilizes the XGBoost to construct two regression equations. GENIE3 is implemented by the RF and Non-linear ODEs inference algorithm is based on the XGBoost. MMFGRN constructs one XGBoost model and two LightGBM models to process steady-state and time-series gene expression data for model fusion.

The running time of the different methods on the DREAM4 *in silico* size100 and *E.coli* datasets is shown in Supplementary Table S6. Longer running time indicates higher computational complexity of the model. And these measurements are conducted on a computer with Intel Core i7-9750H CPU, clocked at 2.60 GHz and 16 GB memory. Our proposed iLSGRN algorithm spends considerable running time while achieving more accurate prediction performance.

## 5 Discussion

Currently, many computational methods for inference of GRNs have been proposed. In this article, we propose a scalable method based on model fusion to infer GRNs from time-series and steady-state series gene expression data. Our method first calculates the MIC between genes and sets a suitable threshold to exclude the redundant and enough weak gene regulatory relationships. Then, we combine XGBoost and RF to learn the non-linear ODEs model and fuse the importance scores. The experimental results on the DREAM4 and *E.coli* datasets show that our method effectively eliminates large redundant gene expression information and improves the accuracy of inferring regulatory relationships.

Compared with other model fusion methods, such as MMFGRN, our method utilizes MIC in the regulatory gene recognition algorithm to remove massive redundant regulatory links in large-scale GRNs. It fundamentally reduces the influence of redundant and enough weak genes on identifying GRNs. For the coexistence of time-series data and steady-state series data, the non-linear ODEs model can more precisely simulate the dynamic characteristics between genes and retain the information in steady-state series data. Moreover, the non-linear ODEs model can comprehensively deal with the non-linear relationships in GRNs. Meanwhile, we integrate XGBoost with RF machine-learning model, i.e. Boost and bagging, to obtain more accurate GRN.

Although our method has improved the prediction accuracy compared to other state-of-the-art methods, there are still some limitations. We only tested the validity of the proposed method on the simulated gene expression dataset of DREAM4 and the real gene expression dataset of *E.coli*. The inference methods obtained low AUPR results on the *E.coli* dataset, which may be due to the sparsity of the GRNs. The real biological datasets only contain steady-state series gene expression data and lack time-series data, which may affect the performance of the developed method. We will further extend the proposed approach on more comprehensive real gene expression datasets in the future. Moreover, this study did not consider the batch effects of gene expression data, which may affect the calculation of the MIC or feature importance assessment. The feature fusion algorithm will increase the computational complexity and time cost of the proposed method due to the training process of XGBoost and RF models. Therefore, developing a faster and more robust algorithm is one of the future directions.

## Supplementary Material

btad619_Supplementary_Data
