# Supplementary material for: iLSGRN: inference of large-scale gene regulatory networks based on multi-model fusion
Source: Bioinformatics. 2023 Oct 18;39(10):btad619. doi: 10.1093/bioinformatics/btad619 (PMC10589915; doi:10.1093/bioinformatics/btad619)
Supplement: btad619_Supplementary_Data [file btad619_supplementary_data.docx]

**iLSGRN: Inference of large-Scale Gene Regulatory Networks based on multi-model fusion**

Supplementary data

Yiming Wu^1^, Bing Qian^1^, Anqi Wang^2^, Heng Dong^1^, Enqiang Zhu^3,*^, Baoshan Ma^1,*^

^1^ School of Information Science and Technology, Dalian Maritime University, Dalian 116026, China

^2^ Department of Statistics and Actuarial Science, The University of Hong Kong, Hong Kong 999077, China

^3^ Institution of Computing Science and Technology, Guangzhou University, Guangzhou 510006, China

*To whom correspondence should be addressed.

1 Supplementary figures

Figure S1: **Distribution of the maximal information coefficients for each sub-network of DREAM4 in silico size100.**

Figure S2: **AUROC curves of iLSGRN on DREAM4** **in silico size100 dataset.**

Figure S3: **Cluster Bar Chart of the results for various methods on each subset of the DREAM4 in silico size100**

Figure S4: **Distribution of the maximal information coefficients for each sub-network of *Escherichia coli*.**

Figure S5: **AUROC curves of iLSGRN on *Escherichia coli*.**

Figure S6: **Cluster Bar Chart of the results on the *Escherichia coli* dataset.**

Figure S7: **Boxplot of overall score with threshold for *Escherichia coli* dataset.**

Figure S8: **Cluster Bar Chart of cross-validation results on the *Escherichia coli* dataset.**


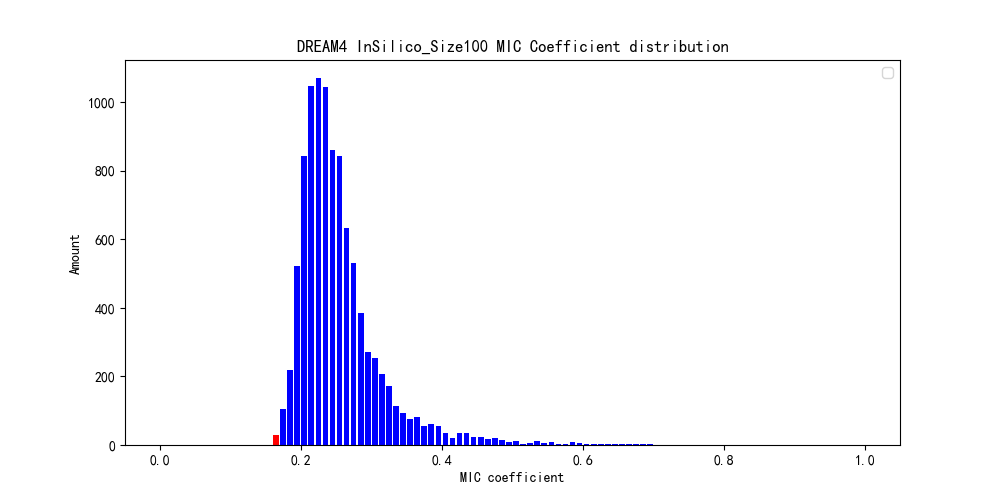


Net1


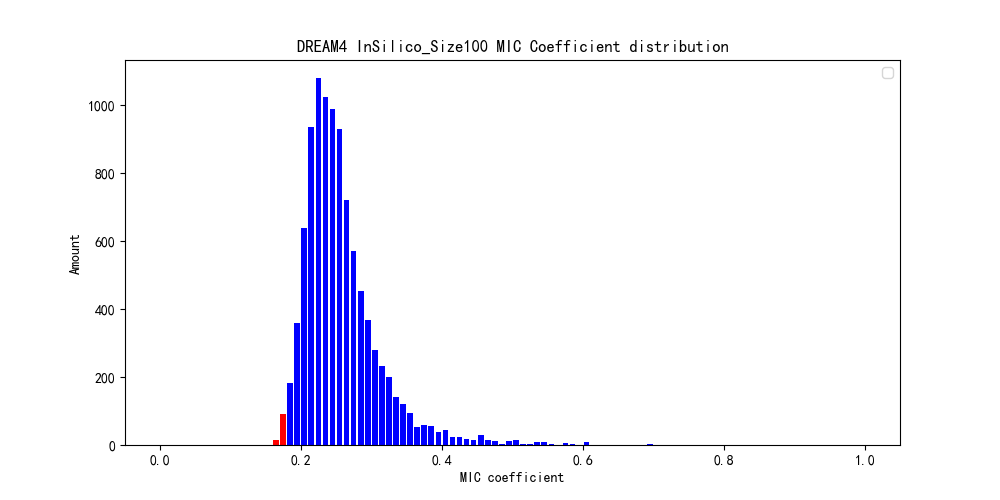


Net2


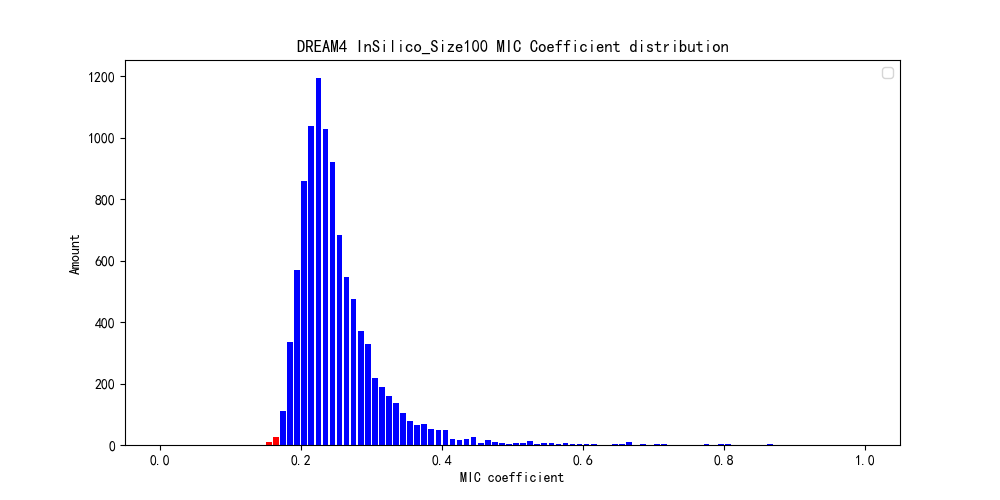


Net3


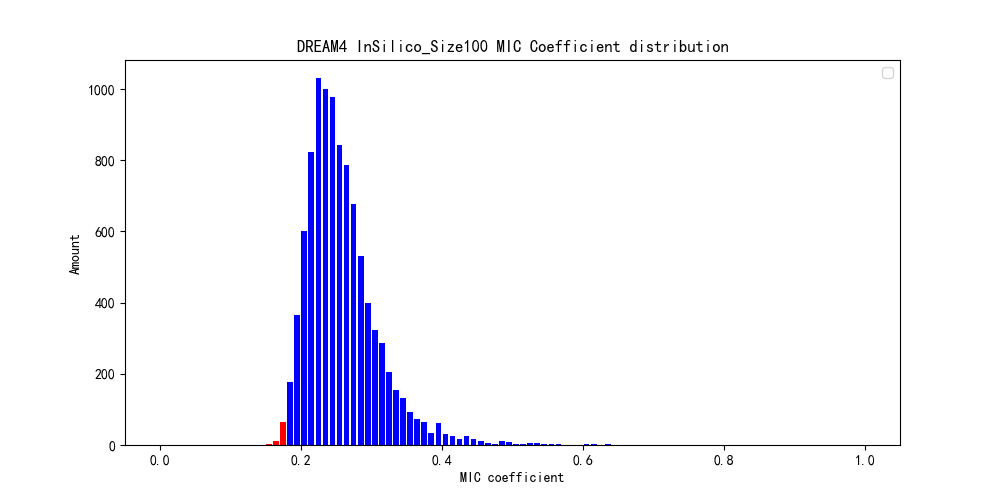


Net4


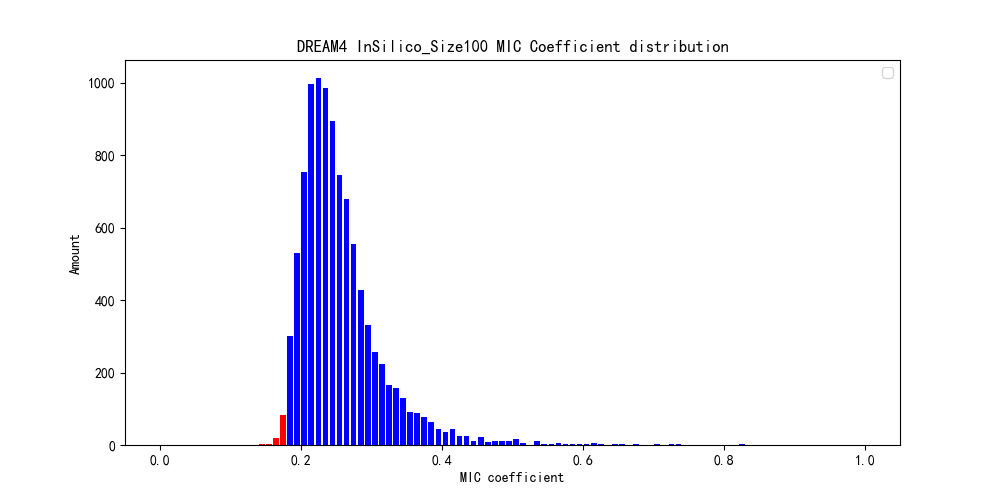


Net5

Figure S1: **Distribution of the** **maximal information coefficients for each sub-network of DREAM4 in silico size100.**

We calculated the maximal information coefficients between genes using the gene expression data of DREAM4 in silico size100 dataset and reported the number of maximal information coefficients at each interval in a histogram. The horizontal coordinate is the value of the maximal information coefficient, and the vertical coordinate is the statistical number of maximal information coefficients in a specific range. The red column denotes the number of edges without regulatory relationships and the blue column denotes the number of edges with potential regulatory relationships. From the histogram, we can observe the distribution of the maximal information coefficient between genes of DREAM4 in silico size100 dataset.


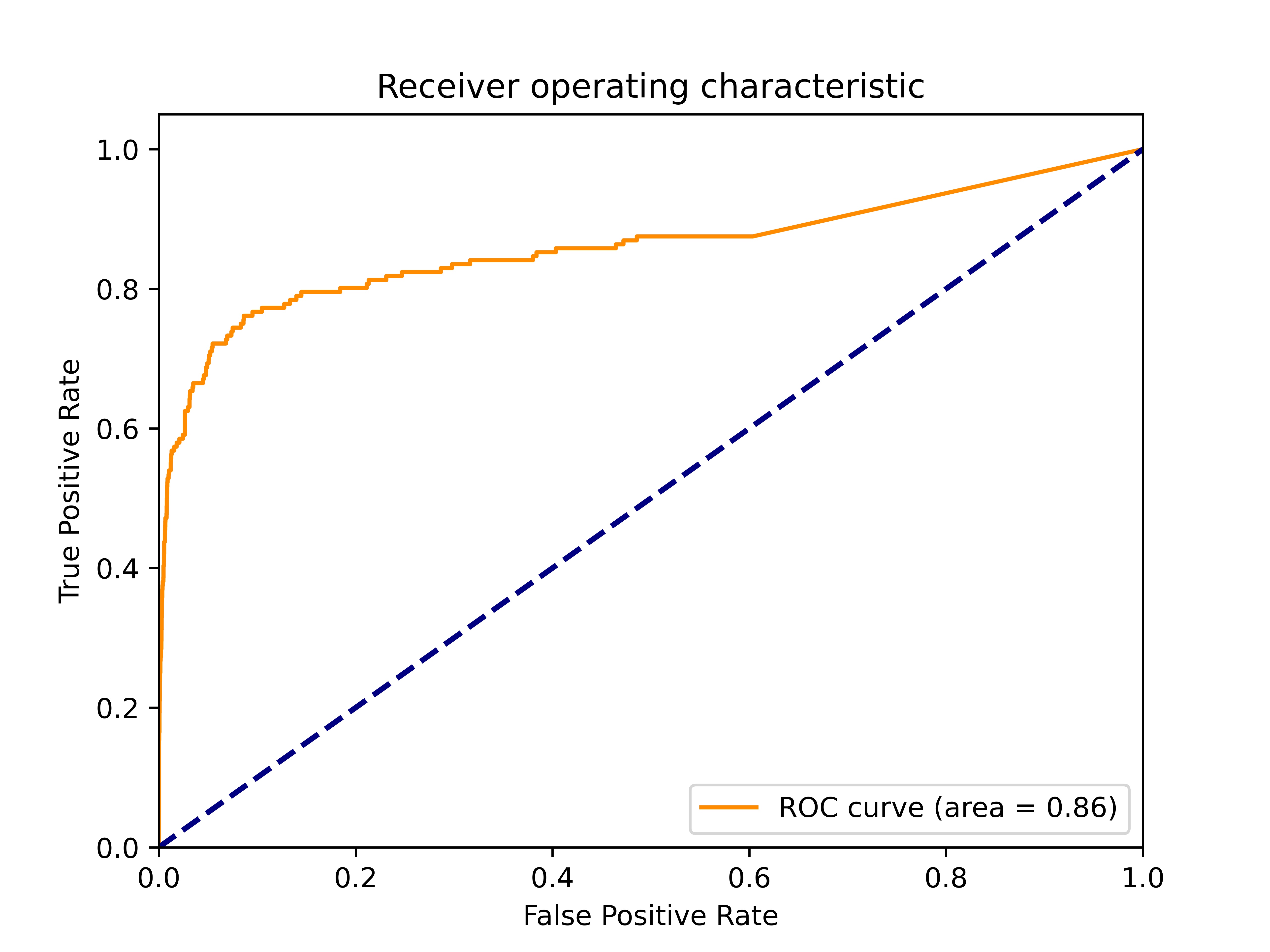

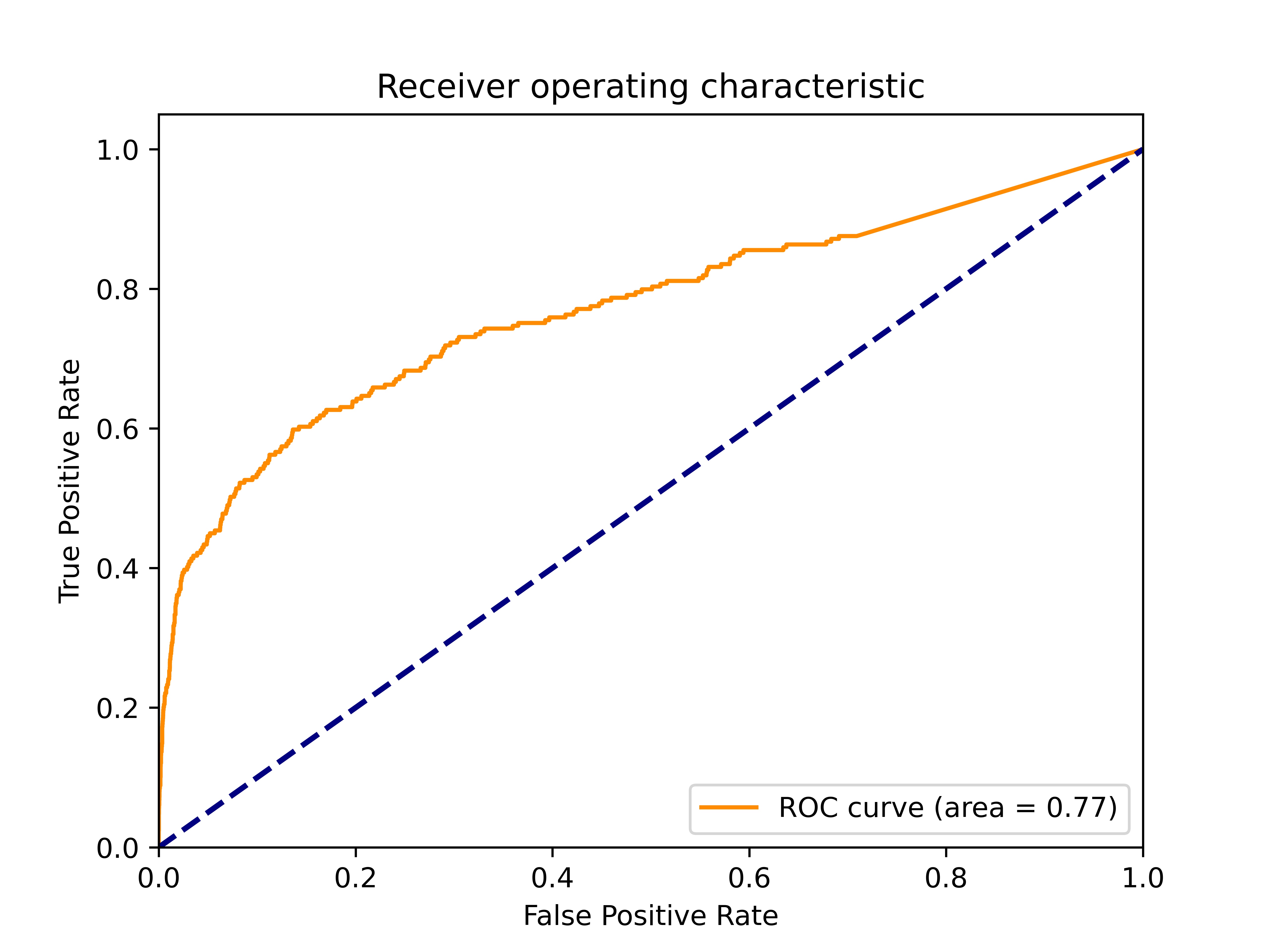


Net1 Net2


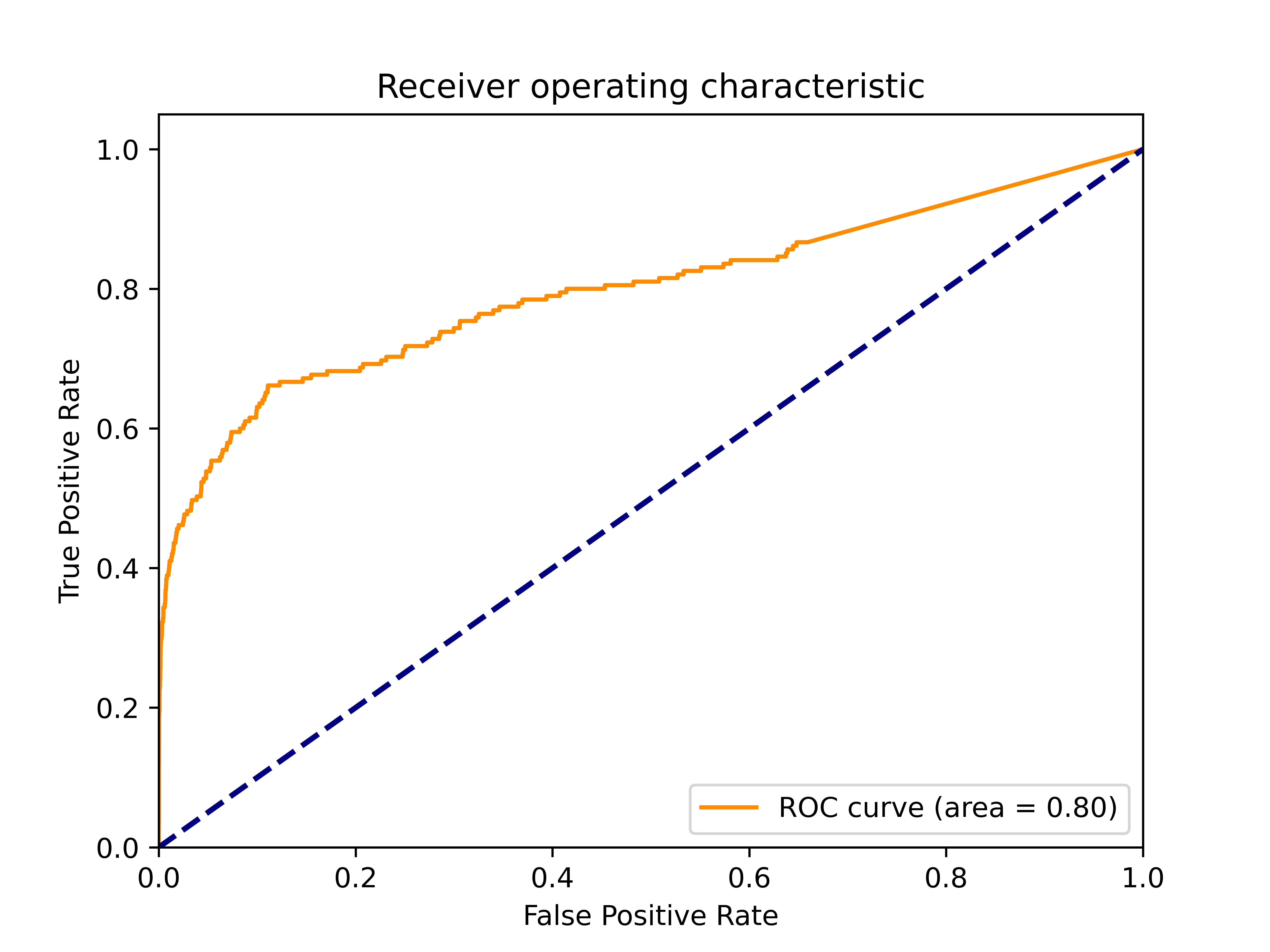

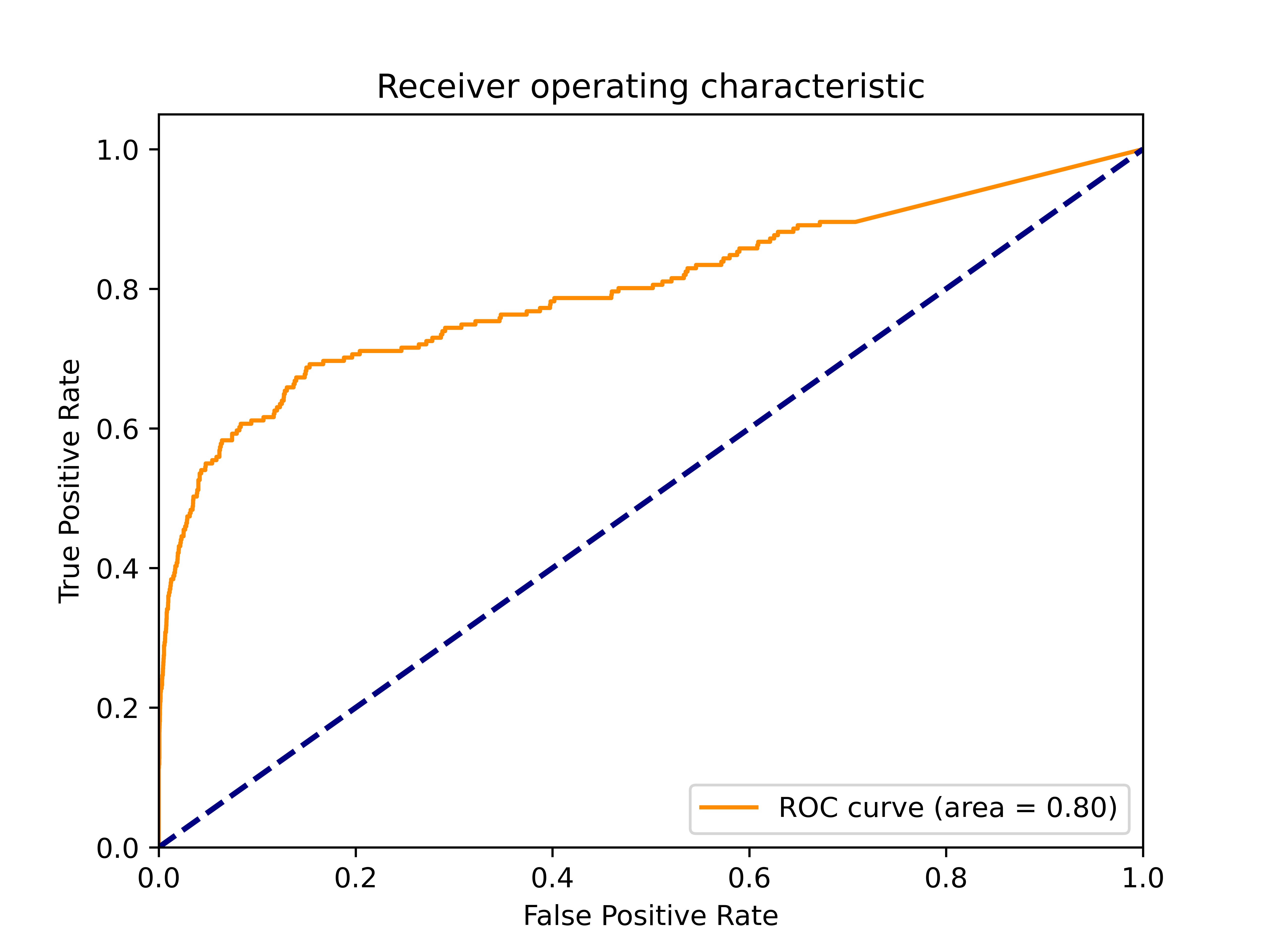


Net3 Net4


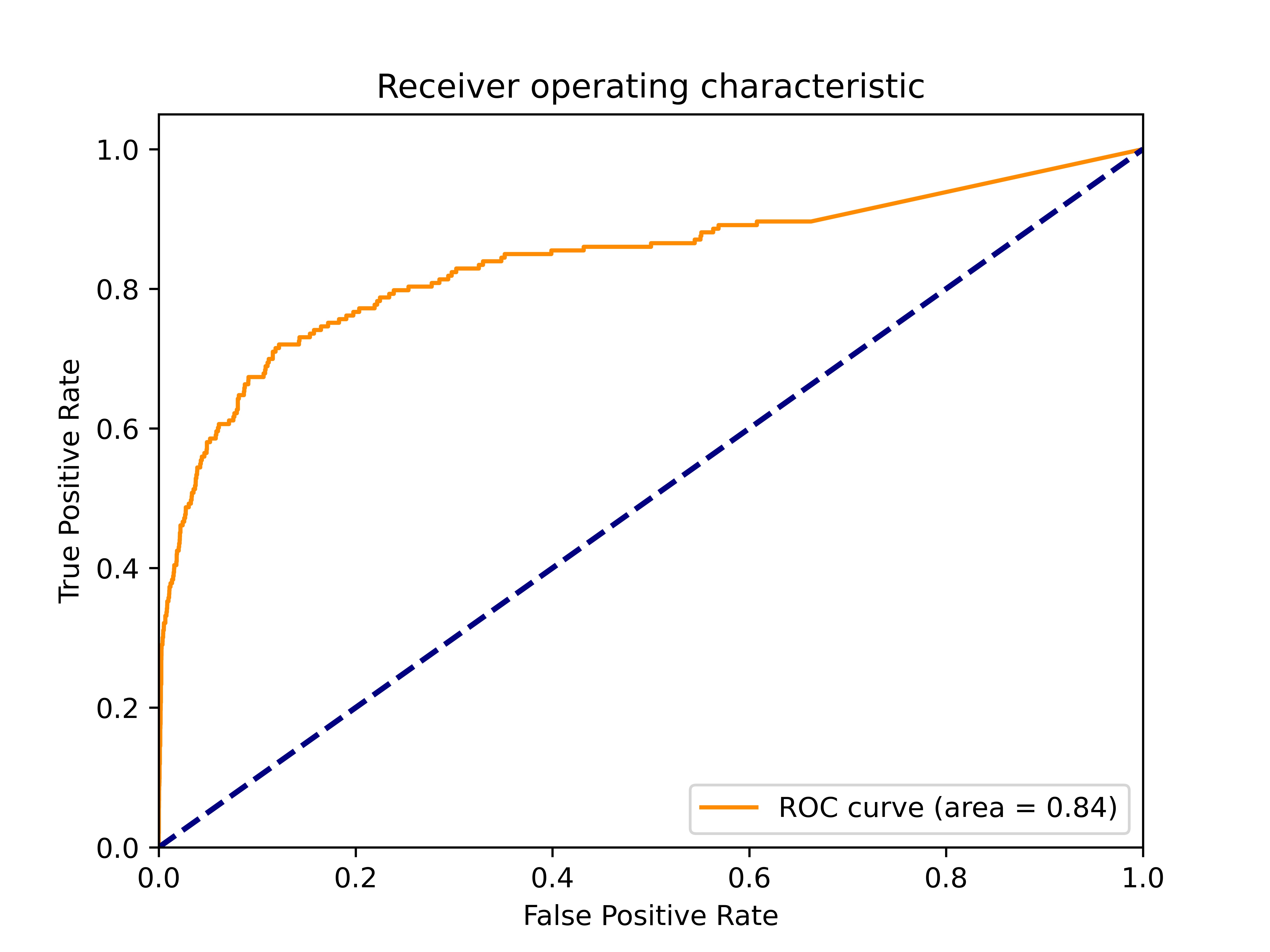


Net5

Figure S2: **AUROC curves of iLSGRN on DREAM4 in silico size100 dataset.**

We plotted the AUROC curves of the iLSGRN algorithm for each sub-network in DREAM4 in silico size100 dataset. The AUROC values from network 1 to network 5 are 0.86, 0.77, 0.80, 0.80, and 0.84.


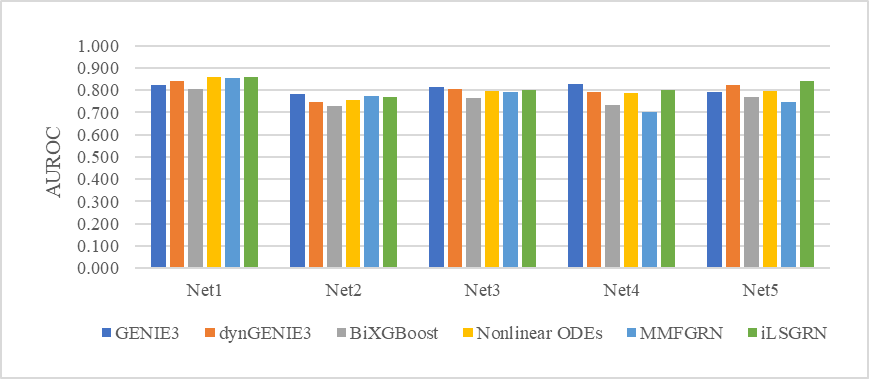


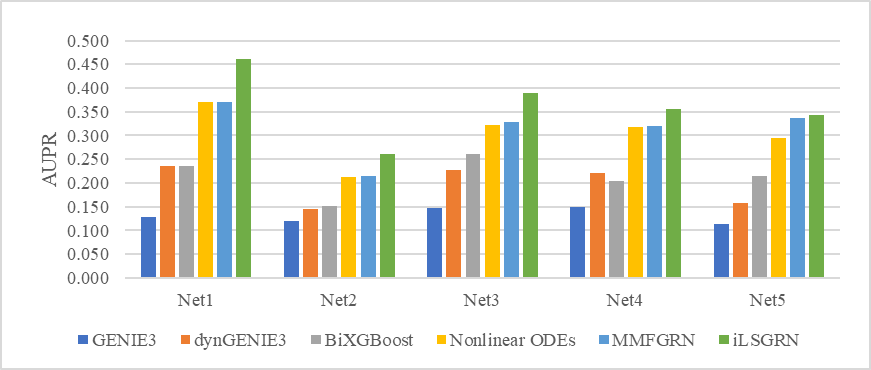


**
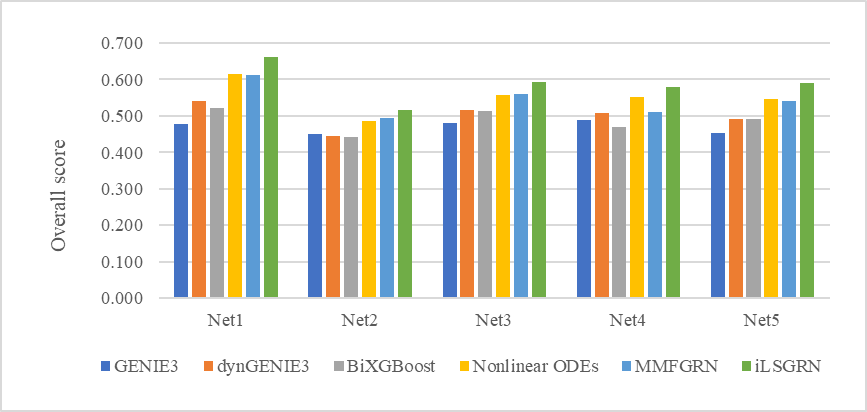
**

Figure S3: **Cluster Bar Chart of the results for various methods on each subset of the DREAM4 in silico size100**

The figure shows the AUROC results, AUPR results, and the overall score results for each method of comparison experiment on the DREAM4 in silico size100 dataset, respectively.


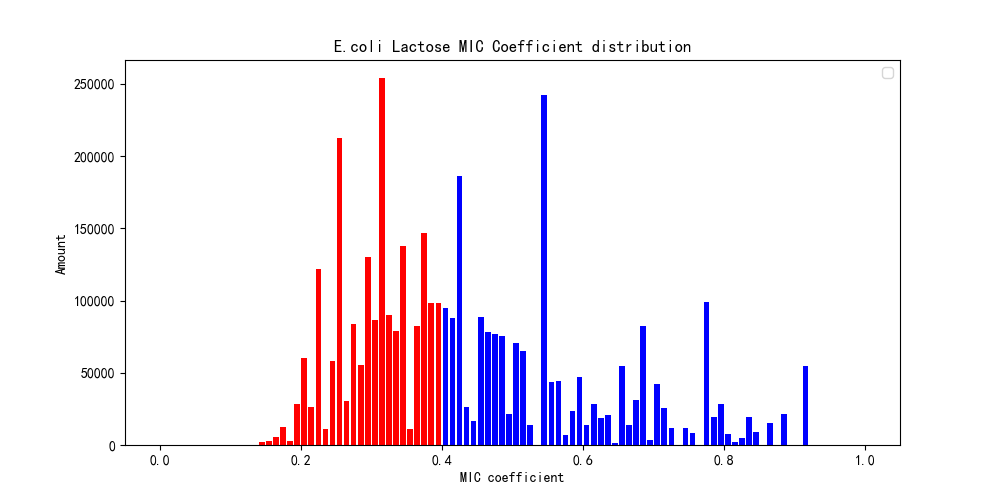


Cold stress


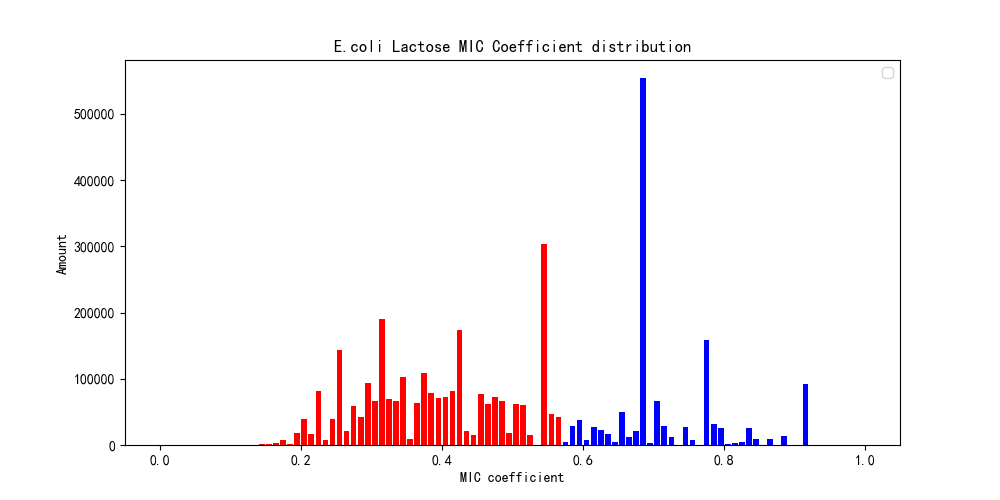


Heat stress


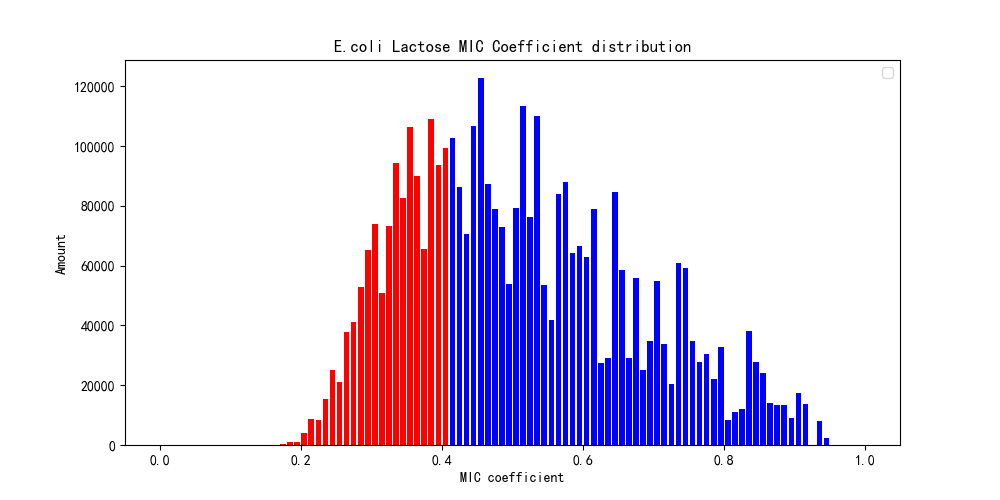


Oxidative stress


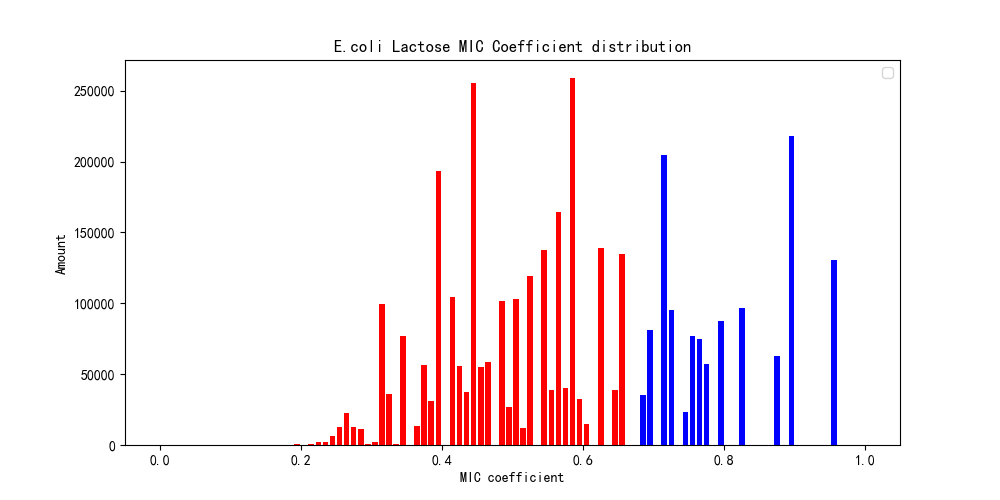


Lactose

Figure S4: **Distribution of the maximal information coefficients for each sub-network of *Escherichia coli*.**

We calculated the maximal information coefficients between genes using the gene expression data of *Escherichia coli* dataset and reported the number of maximal information coefficients at each interval in a histogram. The horizontal coordinate is the value of the maximal information coefficient, and the vertical coordinate is the statistical number of maximal information coefficients in a specific range. The red column denotes the number of edges without regulatory relationships and the blue column denotes the number of edges with potential regulatory relationships. From the histogram, we can observe the distribution of the maximal information coefficient between genes of *Escherichia coli* dataset.


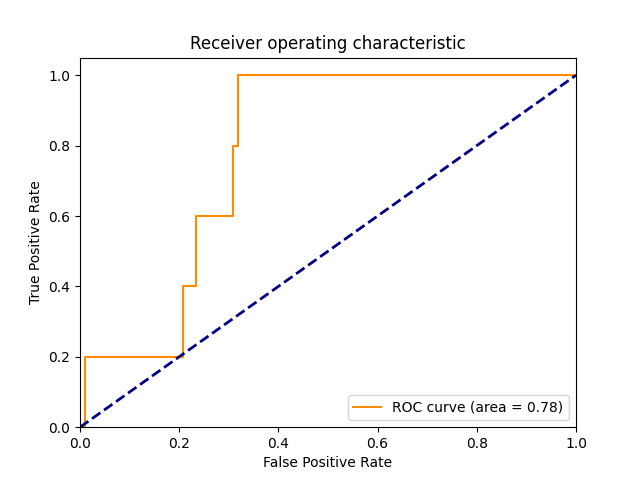

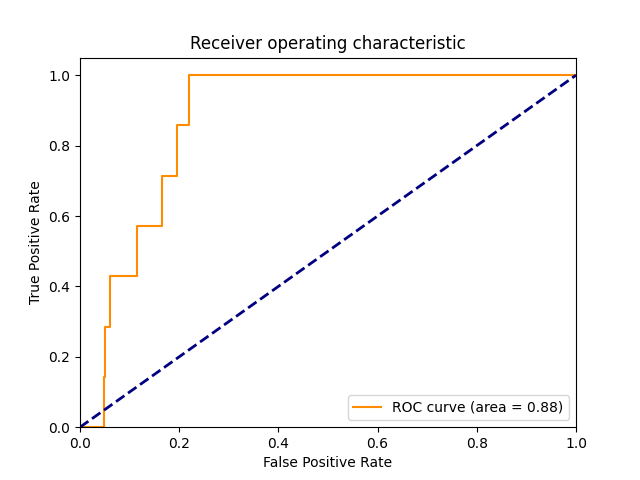


Cold stress Heat stress


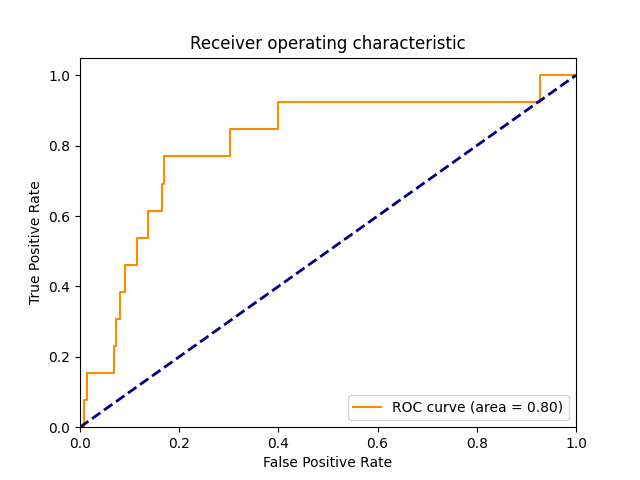

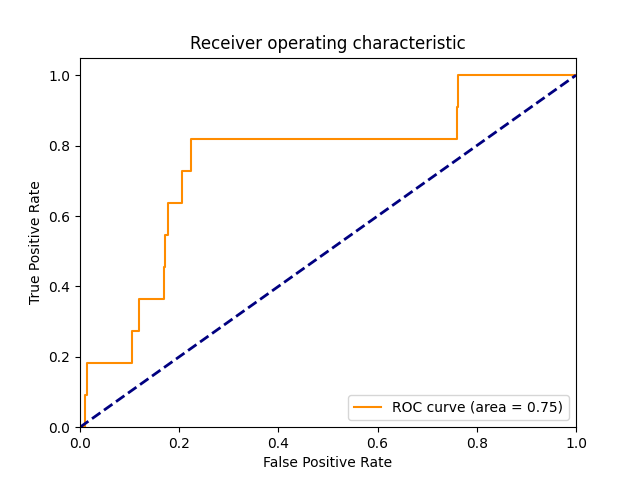


Oxidative stress Lactose

Figure S5: **AUROC curves of iLSGRN on *Escherichia coli*.**

We plotted the AUROC curves of the iLSGRN algorithm for each sub-data set in *Escherichia coli* dataset. The AUROC was 0.78 for Cold stress, 0.88 for Heat stress, 0.80 for Oxidative stress, and 0.75 for Lactose.


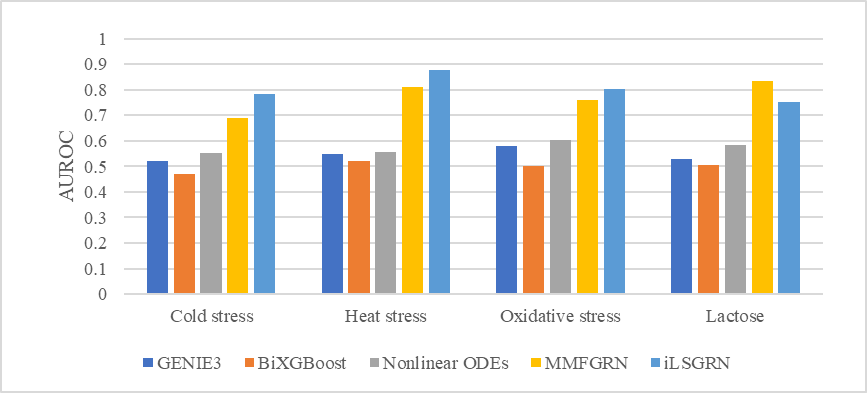


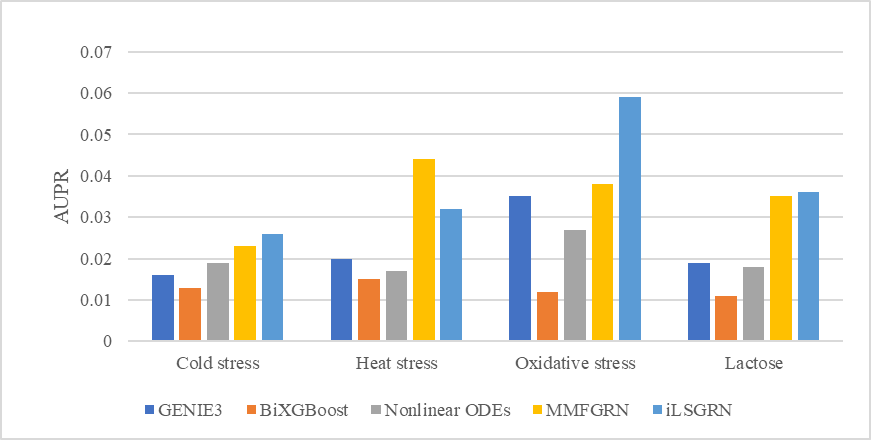


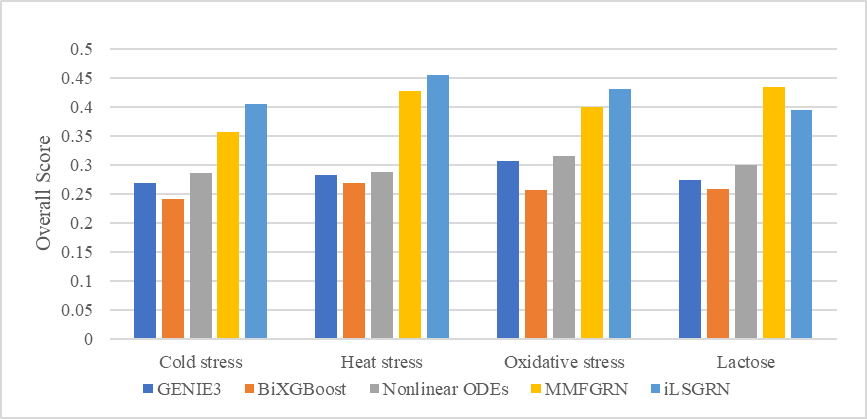


Figure S6: **Cluster Bar Chart of the results on the *Escherichia coli* dataset.**

The figure shows the AUROC results, AUPR results, and the overall score results for each method of comparison experiment on the *Escherichia coli* dataset, respectively.


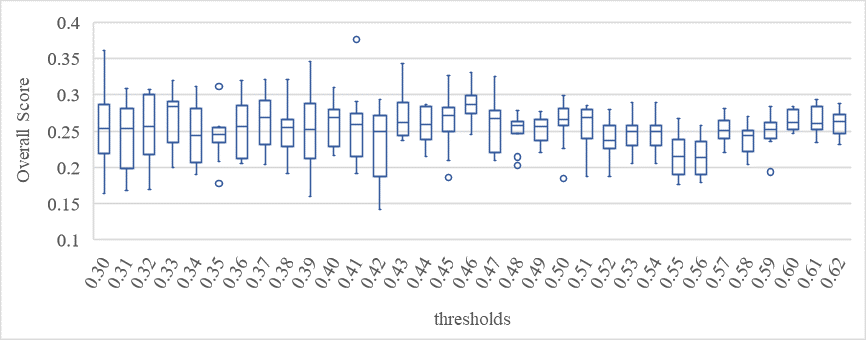


Cold stress 1


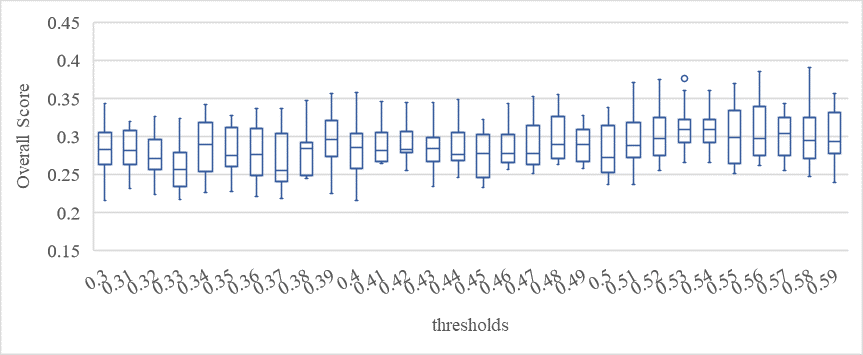


Cold stress 2


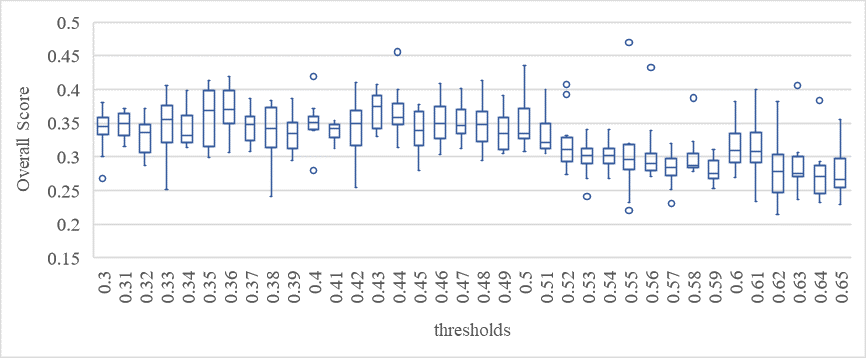


Heat stress 1


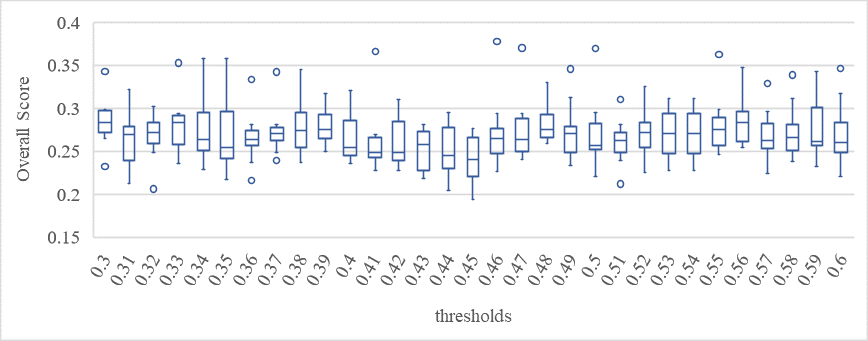


Heat stress 2


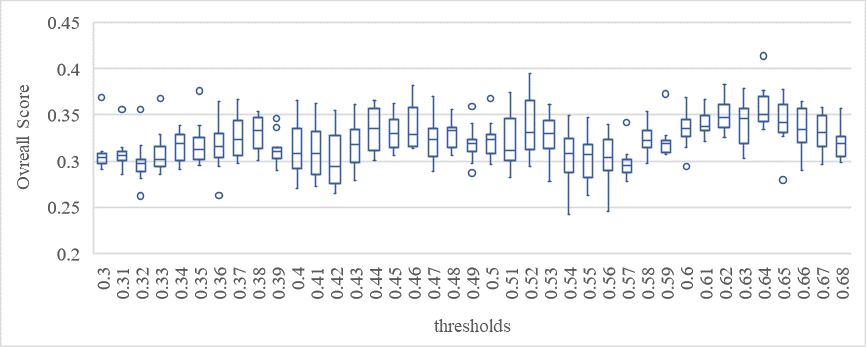


Oxidative stress 1


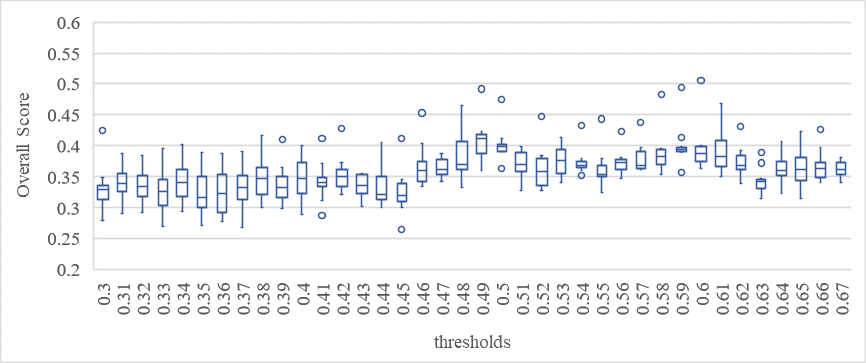


Oxidative stress 2


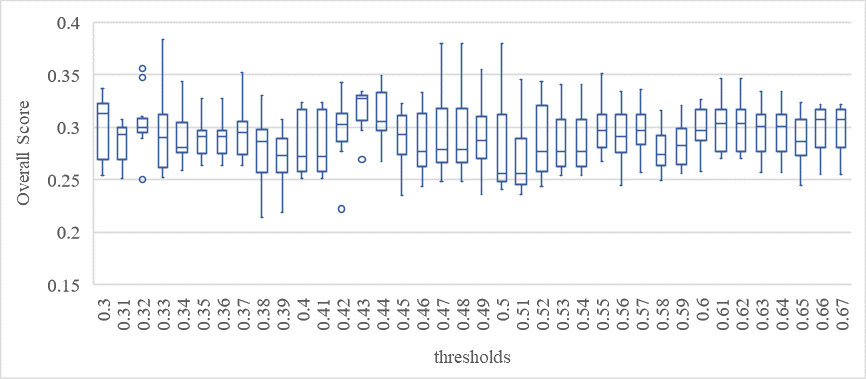


Lactose 1


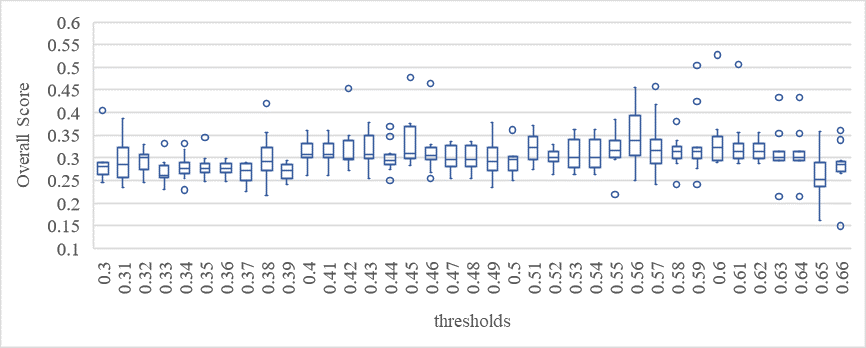


Lactose 2

Figure S7: **Boxplot of overall score with threshold for *Escherichia coli* dataset.**

We conducted twofold cross-validation experiments for the iLSGRN algorithm on the *Escherichia coli* dataset. Taking the Cold stress sub-dataset as an example, we randomly divided it into two equal parts: Cold stress 1 and Cold stress 2. The horizontal axis represents different thresholds, and the vertical axis represents the overall scores. Each box summarizes the overall scores corresponding to 10 different learning rates. We use grid search method to optimize two key parameters in iLSGRN(threshold and the learning rate in XGBoost). Specifically, we set 10 different learning rates of XGBoost and 30 different thresholds of MIC on one training set, conducted a total of 300 experiments and recorded the overall score for each experiment. Next the proposed model with the optimal parameters was applied on the test set. Finally, we switched the training set and test set and repeated the above-mentioned steps.


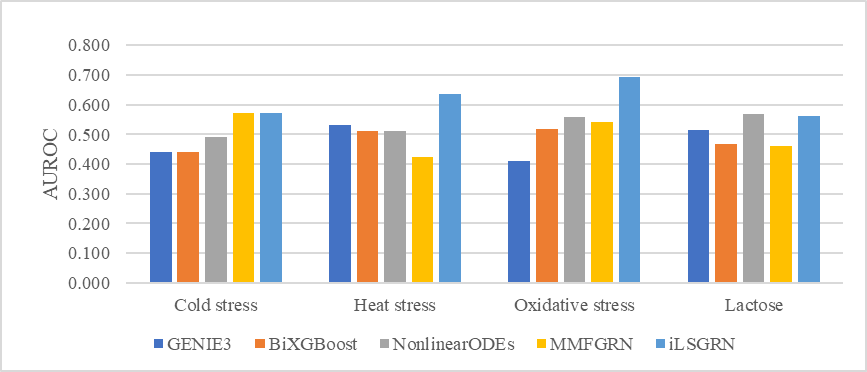


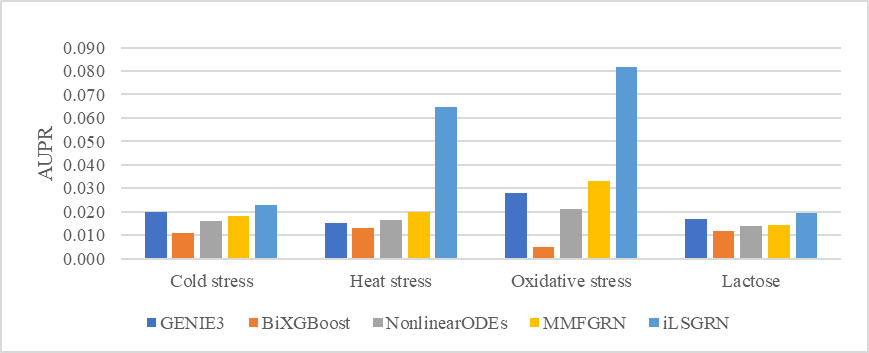


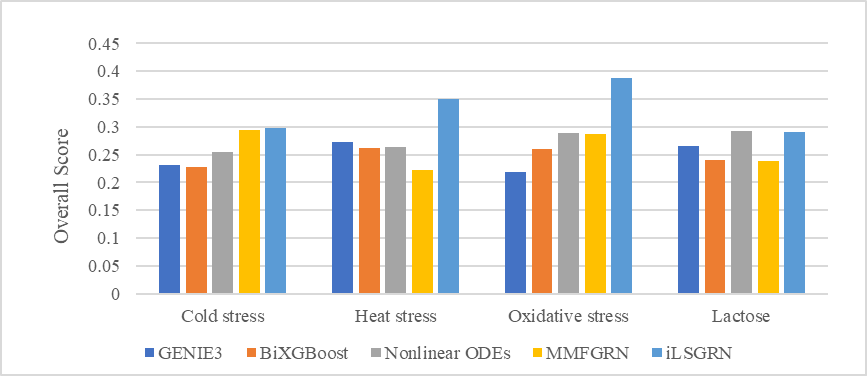


Figure S8: **Cluster Bar Chart of cross-validation results** **on the *Escherichia coli* dataset.**

The figure shows the AUROC results, AUPR results and the overall score results of the cross-validation experiment for each method on the *Escherichia coli* dataset, respectively.

2 Supplementary Tables

Table S1: **Parameters of iLSGRN on the DREAM4 in silico size100 dataset**

Table S2: **EPR for each method on the DREAM4 in silico size100 dataset**

Table S3: **Parameters of iLSGRN on the *Escherichia coli* dataset**

Table S4: **EPR for each method on the *Escherichia coli* dataset**

Table S5: **Parameters of iLSGRN in *Escherichia coli* cross-validation experiments**

Table S6: **The average running time of each method**

Table S1: **Parameters of iLSGRN on the DREAM4 in silico size100 dataset**

| Network | DREAM4 InSilico_Size100 | |
| --- | --- | --- |
|  | learning_rate | threshold |
| Net1 | 0.012 | 0.150 |
| Net2 | 0.012 | 0.177 |
| Net3 | 0.012 | 0.168 |
| Net4 | 0.012 | 0.177 |
| Net5 | 0.012 | 0.173 |

Table S2: **EPR for each method on the DREAM4 in silico size100 dataset**

| Methods | Netwrok1 | Netwrok2 | Netwrok3 | Netwrok4 | Netwrok5 |
| --- | --- | --- | --- | --- | --- |
| GENIE3 | 7.67 | 3.51 | 7.55 | 4.67 | 6.38 |
| dynGENIE3 | 14.38 | 6.55 | 10.93 | 10.67 | 8.77 |
| BiXGBoost | 12.14 | 4.31 | 11.46 | 7.78 | 6.64 |
| NonlinearODEs | 24.29 | 11.98 | 19.79 | 17.57 | 16.74 |
| MMFGRN | 23.94 | 11.87 | 16.66 | 13.12 | 14.88 |
| iLSGRN | 29.08 | 13.57 | 20.83 | 18.01 | 19.40 |

This table shows the EPR results for each method on the DREAM4 in silico size100 dataset, and indicates that our method achieves the highest EPR values on all sub-networks.

Table S3: **Parameters of iLSGRN on the *Escherichia coli* dataset**

| Network | *Escherichia coli* | |
| --- | --- | --- |
|  | learning_rate | threshold |
| Cold stress | 0.85000 | 0.394 |
| Heat stress | 0.55300 | 0.570 |
| Oxidative stress | 0.77725 | 0.410 |
| Lactose | 0.35100 | 0.660 |

Table S4: **EPR for each method on the *Escherichia coli* dataset**

| Methods | Cold stress | Heat stress | Oxidative stress | Lactose |
| --- | --- | --- | --- | --- |
| GENIE3 | 0 | 0 | 0 | 0 |
| dynGENIE3 | 0 | 0 | 3.67 | 3.67 |
| BiXGBoost | 0 | 3.67 | 0 | 3.67 |
| NonlinearODEs | 0 | 0 | 3.67 | 0 |
| MMFGRN | 0 | 3.67 | 3.67 | 7.34 |
| iLSGRN | 3.67 | 0 | 7.34 | 7.34 |

This table shows the EPR results for each method on the *Escherichia coli* dataset. The iLSGRN obtains the highest EPR values on Cold stress, Oxidative stress, and the same EPR value as MMFGRN on Lactose.

Table S5: **Parameters of iLSGRN in *Escherichia coli* cross-validation experiments**

| *Escherichia coli* | | | | | |
| --- | --- | --- | --- | --- | --- |
| Network | learning_rate | threshold | Network | learning_rate | threshold |
| Cold stress 1 | 0.02 | 0.58 | Cold stress 2 | 0.01 | 0.41 |
| Heat stress 1 | 0.01 | 0.46 | Heat stress 2 | 0.01 | 0.55 |
| Oxidative stress 1 | 0.01 | 0.60 | Oxidative stress 2 | 0.01 | 0.64 |
| Lactose 1 | 0.06 | 0.60 | Lactose 2 | 0.01 | 0.33 |

Table S6: **The average running time of each method**

| Methods | DREAM4 in silico size100 | *Escherichia coli* |
| --- | --- | --- |
| GENIE3 | 117s | 33min |
| BiXGBoost | 176s | 12h |
| Nonlinear ODEs | 56s | 7min |
| MMFGRN | 156s | 7h |
| iLSGRN | 148s | 50min |

We investigated the running time of each method on the DREAM4 in silico size100 and *Escherichia coli* datasets. For the experiment on DREAM4 in silico size100, we averaged the running time of each method on five subnetworks. Similarly, for the experiment on *Escherichia coli*, we averaged the running time of each method on four sub-datasets.
